# Early Adverse Neonatal Outcomes Among Women With Diabetes in Pregnancy in Uganda: Incidence, Distribution and Associated Factors in a Multicenter Prospective Cohort Study

**DOI:** 10.1002/hsr2.73404

**Published:** 2026-10-16

**Authors:** Sharifah Namutebi, Marie Pascaline Sabine Ishimwe, Musa Kasujja, Theodore Nteziyaremye, Silver Bagambe, Ibrahim Bwaga, Adam Damulira, Peter Okello, Asanairi Baluku, Theoneste Hakizimana

**Affiliations:** ^1^ Department of Obstetrics and Gynecology Kampala International University Ishaka Uganda; ^2^ Department of Pediatrics and Child Health Kampala International University Ishaka Uganda; ^3^ Department of Sciences University of Rwanda Kigali Rwanda

**Keywords:** adverse neonatal outcomes, diabetes in pregnancy, macrosomia, neonatal hypoglycemia, prospective cohort, respiratory distress, Uganda

## Abstract

**Background and Aims:**

Diabetes in pregnancy increases neonatal metabolic, respiratory, and growth‐related complications, but prospective Ugandan evidence is limited. We determined the incidence and distribution of early adverse neonatal outcomes and assessed associated maternal factors.

**Methods:**

We conducted a multicentre prospective cohort study at Jinja, Hoima, and Lira Regional Referral Hospitals in Uganda. Pregnant women with diabetes were enrolled at admission for delivery, and their newborns were followed until hospital discharge or 7 days of life, whichever occurred first. The composite outcome comprised macrosomia, 5‐min Apgar score < 7, neonatal hypoglycemia, respiratory distress, or early neonatal death. Cumulative incidence was reported with a 95% confidence interval (CI). Modified Poisson regression with robust standard errors was used to estimate crude and adjusted risk ratios.

**Results:**

The analysis included 145 mother–newborn pairs. Overall, 102 newborns experienced at least one early adverse neonatal outcome, corresponding to a cumulative incidence of 70.3% (95% CI: 62.2%–77.6%). Macrosomia occurred in 55/145 newborns (37.9%), 5‐min Apgar score < 7 in 51/145 (35.2%), neonatal hypoglycemia in 38/145 (26.2%), respiratory distress in 31/145 (21.4%), and early neonatal death in 21/145 (14.5%). Neonatal‐unit admission, reported separately as a healthcare‐utilization indicator, occurred in 40/145 newborns (27.6%). After adjustment, women reporting no exercise had a lower risk of the composite outcome than those reporting exercise (adjusted risk ratio 0.68, 95% CI: 0.51–0.91; *p* = 0.009). Maternal body mass index, residence, and antenatal‐care attendance were not clearly associated with the outcome.

**Conclusion:**

Early adverse neonatal outcomes were common among newborns of women with diabetes in pregnancy. The unexpected association with reported exercise may reflect exposure misclassification, reverse causation, or residual confounding and should not be interpreted as evidence that avoiding exercise is beneficial. Strengthened glycemic monitoring, delivery planning, and preparedness for neonatal metabolic and respiratory complications are warranted.

**Abbreviations**
ANC: antenatal care
aRR: adjusted risk ratio
CGM: continuous glucose monitoring
CI: confidence interval
cRR: crude risk ratio
DM: diabetes mellitus
GDM: gestational diabetes mellitus
GWG: gestational weight gain
HbA1c: hemoglobin A1c
HRRH: Hoima regional referral hospital
JRRH: Jinja regional referral hospital
KIU: Kampala international university
LGA: large for gestational age
LMICs: low‐ and middle‐income countries
LRRH: Lira regional referral hospital
NICE: national institute for health and care excellence
NICU: neonatal intensive care unit
REC: research ethics committee
UGX: Uganda shillings

## 1 Introduction

Diabetes mellitus in pregnancy includes pre‐existing type 1 or type 2 diabetes and hyperglycemia first detected during pregnancy. Gestational diabetes mellitus (GDM) refers to hyperglycemia first diagnosed during pregnancy that does not meet the diagnostic thresholds for overt diabetes [1, 2]. Although these conditions differ in their onset, severity, and management, both expose the fetus and newborn to complications associated with maternal hyperglycemia [2, 3].

Diabetes in pregnancy is an increasing global public‐health concern. Using IADPSG diagnostic criteria, the standardized global prevalence of GDM was estimated at 14.0% in 2021, although substantial regional variation was observed [4]. Its prevalence is influenced by maternal age, obesity, genetic susceptibility, population characteristics, screening practices, and diagnostic thresholds [2, 4]. Differences in diagnostic practices are particularly important because limited testing may fail to identify clinically important fasting or post‐load hyperglycemia [1, 5].

Maternal glucose crosses the placenta and stimulates fetal insulin production. Sustained fetal hyperinsulinemia promotes excessive fat deposition and fetal growth and may alter pulmonary and metabolic adaptation after birth [2, 3]. The Hyperglycemia and Adverse Pregnancy Outcome study demonstrated continuous associations between increasing maternal glucose concentrations and adverse pregnancy outcomes, even at glucose levels below those used to diagnose overt diabetes [6]. These findings informed the IADPSG recommendations, which classify hyperglycemia in pregnancy using fasting, 1‐h and 2‐h plasma glucose concentrations obtained during an oral glucose‐tolerance test [1].

Newborns of women with diabetes are at increased risk of macrosomia, neonatal hypoglycemia, respiratory distress, low 5‐min Apgar scores, birth trauma, and perinatal death [3, 7, 8]. A population‐based study of more than 700,000 births reported increased risks of macrosomia, respiratory distress, preterm birth, and birth trauma among pregnancies complicated by GDM [7]. A meta‐analysis similarly found that both gestational and pre‐existing diabetes were associated with an increased risk of neonatal respiratory distress syndrome [9]. These outcomes may occur concurrently; therefore, examining both the overall composite burden and the distribution of individual outcomes provides a more complete description of neonatal morbidity.

The occurrence of adverse neonatal outcomes is influenced by factors beyond hyperglycemia alone. Diabetes type, timing of diagnosis, maternal body mass index, gestational age, glycemic control, treatment and coexisting obstetric complications may modify neonatal risk [2, 8, 10]. Elevated pre‐pregnancy body mass index has been associated with macrosomia, low 5‐min Apgar scores, and other adverse neonatal outcomes [10]. Appropriate physical activity may improve maternal glucose regulation and reduce the risk of GDM, although its relationship with neonatal outcomes depends on the type, intensity, duration and timing of activity [11, 12].

Timely identification and management of maternal hyperglycemia can reduce some adverse outcomes. Randomized trials have demonstrated that treating GDM reduces excessive fetal growth and selected pregnancy complications [13, 14]. More recently, treatment of GDM diagnosed before 20 weeks of gestation produced a modest reduction in a composite of adverse neonatal outcomes [15]. However, neonatal complications may still occur despite treatment, and their frequency may vary according to glycemic control, treatment modality, and underlying maternal risk [16].

Evidence from Africa remains limited and heterogeneous. A systematic review found substantial variation in GDM prevalence and diagnostic approaches, with relatively few African countries contributing primary data [17]. A South African cohort demonstrated that GDM influenced fetal growth and neonatal anthropometric measurements, although the observed relationships differed from some findings reported in high‐income settings [18]. In Northwest Ethiopia, composite adverse neonatal outcomes occurred more frequently among newborns of women with GDM than among those without GDM [19].

In Uganda, a prospective cohort found that hyperglycemia in pregnancy was associated with cesarean delivery, large‐for‐gestational‐age birth and neonatal‐unit admission, with the greatest risks observed when both fasting and post‐load glucose concentrations were elevated [5]. Nevertheless, multicentre evidence remains limited regarding the incidence, distribution, and factors associated with early adverse neonatal outcomes among women with diabetes receiving care at regional referral hospitals. Such evidence is required to support risk assessment, delivery planning, and appropriate neonatal preparedness.

Therefore, this study aimed to determine the incidence of early adverse neonatal outcomes among women with diabetes mellitus at Jinja, Hoima, and Lira Regional Referral Hospitals; describe the distribution of specific early adverse neonatal outcomes among their newborns; and identify maternal, obstetric, and clinical factors associated with the composite early adverse neonatal outcome.

## 2 Materials and Methods

### 2.1 Study Design and Setting

We conducted a multicentre, hospital‐based prospective cohort study over 5 months at Jinja, Hoima, and Lira Regional Referral Hospitals in Uganda. Pregnant women with diabetes mellitus were enrolled during admission for delivery and followed through labor and childbirth. Their newborns were subsequently followed from birth until hospital discharge or 7 days of life, whichever occurred first. This design enabled estimation of the cumulative incidence and distribution of early adverse neonatal outcomes and assessment of maternal factors associated with the composite outcome.

Jinja, Hoima, and Lira Regional Referral Hospitals are public tertiary referral facilities serving predominantly eastern, western, and northern Uganda, respectively. They receive patients from urban and rural communities and manage referrals from district hospitals and lower‐level health facilities. Each hospital provides comprehensive maternity, operative obstetric, laboratory, and neonatal‐care services, including management of high‐risk pregnancies and neonatal metabolic and respiratory complications.

The hospitals were purposively selected because they serve geographically distinct populations, manage substantial numbers of high‐risk pregnancies, and have maternity and neonatal services capable of identifying and managing complications among newborns exposed to maternal diabetes. Standardized eligibility criteria, outcome definitions, data‐collection instruments, and follow‐up procedures were applied across the three sites. However, routine diabetes‐management and neonatal‐care practices were not experimentally standardized and may have varied between hospitals. Because recruitment was limited to regional referral hospitals, the findings primarily represent women with diabetes receiving referral‐level obstetric care and may not be generalizable to women managed in lower‐level facilities or community settings.

### 2.2 Study Population

The study population comprised pregnant women with confirmed diabetes mellitus in pregnancy who were admitted for delivery at Jinja, Hoima, or Lira Regional Referral Hospital during the study period. Eligible women were enrolled before delivery and followed through labor and childbirth, after which their newborns were followed until hospital discharge or 7 days of life, whichever occurred first.

### 2.3 Inclusion Criteria

Women were eligible if they had documented pre‐existing diabetes mellitus, overt diabetes first diagnosed during pregnancy, or gestational diabetes mellitus recorded in their antenatal or medical records. Previously undiagnosed women with classic symptoms of hyperglycemia were also eligible if their random blood glucose concentration at admission was ≥ 200 mg/dL. Additional eligibility requirements were admission for delivery at a participating hospital, a live fetus at enrollment, and provision of written informed consent.

### 2.4 Exclusion Criteria

Women were excluded if diabetes could not be confirmed according to the study criteria. This included women without documented diabetes whose random blood glucose concentration was 140–199 mg/dL, because this range was considered screen‐positive but not diagnostic of gestational diabetes. These women were referred to the routine obstetric team for further clinical assessment and confirmatory follow‐up. Women with major coexisting conditions that could independently influence neonatal outcomes, those unable to provide informed consent, and mother–newborn pairs without ascertainable early neonatal‐outcome data were also excluded.

### 2.5 Sample Size Determination

The sample size was determined for the primary objective of estimating the cumulative incidence of early adverse neonatal outcomes among women with diabetes mellitus in pregnancy. The Kish–Leslie single‐population‐proportion formula was applied using an anticipated incidence of 30.0%, derived from the overall incidence reported in a prospective Ethiopian cohort, a 95% confidence level, and 8% absolute precision [19]. This yielded a minimum sample size of 126 mother–newborn pairs. After adding 10% for possible incomplete observations or loss to follow‐up, at least 139 pairs were required. The recruitment target was increased to 150 pairs to improve the precision of the incidence estimate. Of these, 145 had complete maternal exposure and early neonatal‐outcome data and were included in the final analysis. This analytical sample exceeded the minimum required for the primary incidence objective. Analyses of maternal factors associated with the composite outcome were secondary and exploratory; therefore, their estimates were interpreted according to their magnitude and precision rather than statistical significance alone.

### 2.6 Sampling Technique

Consecutive sampling was used at all three hospitals. Labor‐ward admission and antenatal records were reviewed daily to identify pregnant women with documented diabetes mellitus or those requiring diabetes assessment under the study screening procedure. All women who met the eligibility criteria were approached consecutively and enrolled after providing written informed consent. No fixed site quotas were applied; each hospital's contribution reflected the number of eligible women recruited during the study period. The final analytical cohort comprised 145 mother–newborn pairs: 52 from Lira, 47 from Jinja, and 46 from Hoima Regional Referral Hospital. Standardized eligibility criteria, outcome definitions, data‐collection instruments, and follow‐up procedures were applied across the three hospitals. Site‐specific participant contributions and outcome proportions were reported descriptively.

### 2.7 Data Collection Procedure

Potentially eligible women admitted for delivery were identified through labor‐ward registers, antenatal records, and medical histories. Women were approached privately, provided with study information in English or their preferred local language, and enrolled after giving written informed consent. Trained research assistants administered a structured, pretested questionnaire to collect sociodemographic characteristics, obstetric history, antenatal‐care attendance, medical history, diabetes treatment, and self‐reported exercise during pregnancy.

Diabetes status was initially established from antenatal cards or medical records documenting pre‐existing diabetes mellitus, overt diabetes first diagnosed during pregnancy, or gestational diabetes mellitus diagnosed during antenatal care. Previously unscreened women presenting with classic symptoms of hyperglycemia underwent clinical assessment and random capillary blood‐glucose measurement at admission. Under the study protocol, a random blood glucose concentration ≥200 mg/dL in the presence of classic symptoms was considered diagnostic of diabetes and qualified the woman for enrollment. Values of 140–199 mg/dL were considered screen‐positive but not independently diagnostic of gestational diabetes. Women identified solely within this range were not enrolled and were referred to the routine obstetric team for further clinical assessment and confirmatory follow‐up. Random blood glucose was used only as a pragmatic intrapartum case‐finding test because fasting or oral glucose‐tolerance testing was not feasible without delaying the care of women admitted in labor.

Maternal weight and height were measured using standardized equipment, and body mass index was calculated as weight in kilograms divided by height in meters squared. Participants were followed prospectively through labor and delivery. Delivery information and neonatal characteristics were recorded using standardized clinical forms. Birth weight was measured immediately after delivery using a calibrated neonatal weighing scale. Gestational age was determined from the best obstetric estimate and, when reliable menstrual or early‐ultrasound dating was unavailable, assessed using the New Ballard Score.

Newborns were followed from birth until hospital discharge or 7 days of life, whichever occurred first. Neonatal blood glucose was measured by heel‐prick using a point‐of‐care glucometer, and hypoglycemia was defined as a concentration <40 mg/dL. Birth weight, 5‐min Apgar score, respiratory distress, neonatal hypoglycemia, neonatal‐unit admission, and death during the observed follow‐up period were documented through direct clinical assessment and review of hospital records. Newborns who developed complications received routine clinical management and, when indicated, were referred to the pediatric or neonatal‐care team. Completed study forms were reviewed daily for completeness and internal consistency.

### 2.8 Study Variables

The primary analytical outcome was a composite early adverse neonatal outcome, defined as the occurrence of at least one of the following from birth until hospital discharge or 7 days of life, whichever occurred first: macrosomia, 5‐min Apgar score <7, neonatal hypoglycemia, respiratory distress, or neonatal death. Newborns without any component were classified as having no early adverse neonatal outcome.

Macrosomia was defined as birth weight ≥4000 g, and neonatal hypoglycemia as blood glucose <40 mg/dL. Respiratory distress was defined by tachypnea, grunting, nasal flaring, or chest retractions. Neonatal death was defined as death during the observed follow‐up period. Neonatal‐unit admission was reported separately as a healthcare‐utilization indicator and was not included in the composite outcome.

The maternal variables included in the analysis were age, body mass index, religion, education level, residence, antenatal‐care attendance, and self‐reported exercise during pregnancy. Body mass index was categorized as <25.0, 25.0–29.9, or ≥30.0 kg/m^2^. Antenatal‐care attendance was categorized as fewer than four or at least four contacts, while exercise was recorded as a binary self‐reported variable.

### 2.9 Ascertainment of Diabetes Mellitus in Pregnancy

Diabetes in pregnancy was established using documented clinical records or the study's admission‐screening procedure. Women were eligible if their antenatal card or medical records documented pre‐existing diabetes mellitus, overt diabetes first diagnosed during pregnancy, or gestational diabetes mellitus diagnosed during antenatal care. Among women without documented antenatal testing who presented with classic symptoms of hyperglycemia, random blood glucose was measured at admission. A random blood glucose concentration ≥200 mg/dL in the presence of classic symptoms was considered diagnostic of diabetes and qualified the woman for enrollment. Random blood glucose was used in this limited context because it permitted rapid assessment without delaying intrapartum care.

Random blood glucose concentrations of 140–199 mg/dL were considered screen‐positive hyperglycemia requiring further clinical assessment but were not independently diagnostic of gestational diabetes. Women identified solely within this range were not enrolled or included in the analytical cohort; they were referred to the routine obstetric team for appropriate follow‐up and confirmatory evaluation.

### 2.10 Assessment of Early Adverse Neonatal Outcomes

Early adverse neonatal outcomes were assessed prospectively from birth until hospital discharge or 7 days of life, whichever occurred first. Trained research assistants used a standardized checklist and reviewed delivery, postnatal, and neonatal records to document each prespecified outcome.

Birth weight was measured using a calibrated neonatal weighing scale, and macrosomia was defined as birth weight ≥4000 g. Apgar scores were assigned by the attending clinical team at one and 5 min after birth; a 5‐min score <7 was classified as an adverse outcome. Neonatal blood glucose was measured from a heel‐prick sample using a point‐of‐care glucometer, with hypoglycemia defined as a concentration <40 mg/dL. Respiratory distress was identified clinically by tachypnea, grunting, nasal flaring or chest retractions. Early neonatal death was defined as death within 7 days after birth.

The composite outcome was classified as present when a newborn experienced at least one of these five outcomes. Because individual components were not mutually exclusive, a newborn could contribute to more than one component‐specific estimate but only once to the composite outcome. Neonatal‐unit admission was documented and reported separately because it reflects healthcare utilization and may be influenced by clinical protocols, bed availability, and admission thresholds.

### 2.11 Data Quality Control

Research assistants were trained in the study objectives, eligibility assessment, informed‐consent procedures, standardized questionnaire administration, anthropometric measurements, neonatal‐outcome definitions and follow‐up procedures. The questionnaire and neonatal‐outcome checklist were pretested for 2 weeks among a similar population at Kampala International University Teaching Hospital, and necessary revisions were made before data collection. Standardized eligibility criteria, data‐collection instruments and outcome definitions were applied across the three study sites.

Maternal weight, height, and neonatal birth weight were measured using calibrated equipment. Maternal and neonatal blood glucose were assessed using the same standardized finger‐prick and heel‐prick procedures, respectively, and glucometers were operated according to the manufacturer's instructions. The principal investigator supervised data collection, and completed questionnaires and clinical checklists were reviewed daily for completeness and internal consistency. Discrepancies were checked against the source records and corrected before data entry whenever possible. Participants were identified using unique study codes to maintain accurate linkage between maternal and neonatal records while protecting confidentiality.

### 2.12 Data Management and Analysis

Completed questionnaires and clinical follow‐up forms were identified using unique study codes, reviewed for completeness, and securely stored. Data were entered into Microsoft Excel, checked using range, consistency and logic checks, cleaned, and exported to Stata version 14.2 (StataCorp, College Station, TX, USA) for analysis. The final analytical dataset contained 145 mother–newborn pairs with complete data for the variables included in the analysis.

Categorical variables were summarized using frequencies and percentages. Continuous variables were summarized using means and standard deviations when approximately normally distributed or medians and interquartile ranges when skewed. The cumulative incidence of the composite early adverse neonatal outcome was calculated as the proportion of newborns experiencing at least one component outcome and reported with a 95% Wilson confidence interval. Individual outcome components were summarized using frequencies and percentages. Because components were not mutually exclusive, their percentages were not summed. Neonatal‐unit admission was analyzed separately.

The cumulative incidence and distribution of individual early adverse neonatal outcomes were prespecified primary analyses. Assessment of associated maternal factors was a prespecified secondary analysis, while data‐driven variable selection was considered exploratory. Bivariable modified Poisson regression with robust standard errors was used to estimate crude risk ratios (cRRs) and 95% confidence intervals. Variables with *p* < 0.20 at bivariable analysis, together with clinically relevant variables supported by previous evidence, were considered for multivariable analysis. All tests were two‐sided, with a prespecified α level of 0.05. Findings were interpreted primarily according to the magnitude and precision of effect estimates rather than statistical significance alone.

Because the composite outcome was common, multivariable modified Poisson regression with robust standard errors was used to estimate adjusted risk ratios (aRRs) rather than odds ratios. The final model included maternal body mass index, residence, antenatal‐care contacts, and reported exercise status. Multicollinearity was assessed using variance inflation factors. Site‐specific outcome proportions were compared descriptively using Pearson's *χ* ^2^ test; however, the study site was not included in the participant‐level adjusted model.

All tests were two‐sided. Findings were interpreted primarily according to the magnitude and precision of effect estimates rather than statistical significance alone. *p* values were reported as supplementary measures of statistical evidence: *p* < 0.001 for values below 0.001, to three decimal places for values from 0.001 to <0.01, to two decimal places for values ≥0.01 and as *p* > 0.99 when applicable.

This manuscript was prepared in accordance with the Strengthening the Reporting of Observational Studies in Epidemiology reporting guideline for cohort studies.

### 2.13 Ethical Considerations

Ethical approval was obtained from the Kampala International University Research and Ethics Committee (KIU‐REC; approval number KIU‐2022‐140). Administrative permission was obtained from Jinja, Hoima and Lira Regional Referral Hospitals before data collection. The study was conducted in accordance with applicable national ethical requirements and the principles of the Declaration of Helsinki.

Written informed consent was obtained from each participant in English or her preferred local language before enrollment. Participation was voluntary, and women could decline or withdraw without affecting their clinical care. Interviews were conducted privately, and unique study identification codes were used instead of names. Hard‐copy records were stored in locked cabinets, while deidentified electronic data were password‐protected and accessible only to authorized study personnel.

Maternal and neonatal capillary blood sampling was performed using aseptic procedures and involved minimal discomfort. Women and newborns continued to receive routine clinical care throughout the study. Newborns identified with hypoglycemia, respiratory distress, or another complication received appropriate treatment or referral to the pediatric or neonatal‐care team.

## 3 Results

### 3.1 Baseline Characteristics of Participants

Of the 150 enrolled mother–newborn pairs, five did not satisfy the final eligibility or analytical data‐completeness requirements and were excluded. The remaining 145 pairs had complete data required for the primary analyses and constituted the final analytical cohort (Figure 1).

**Figure 1:**
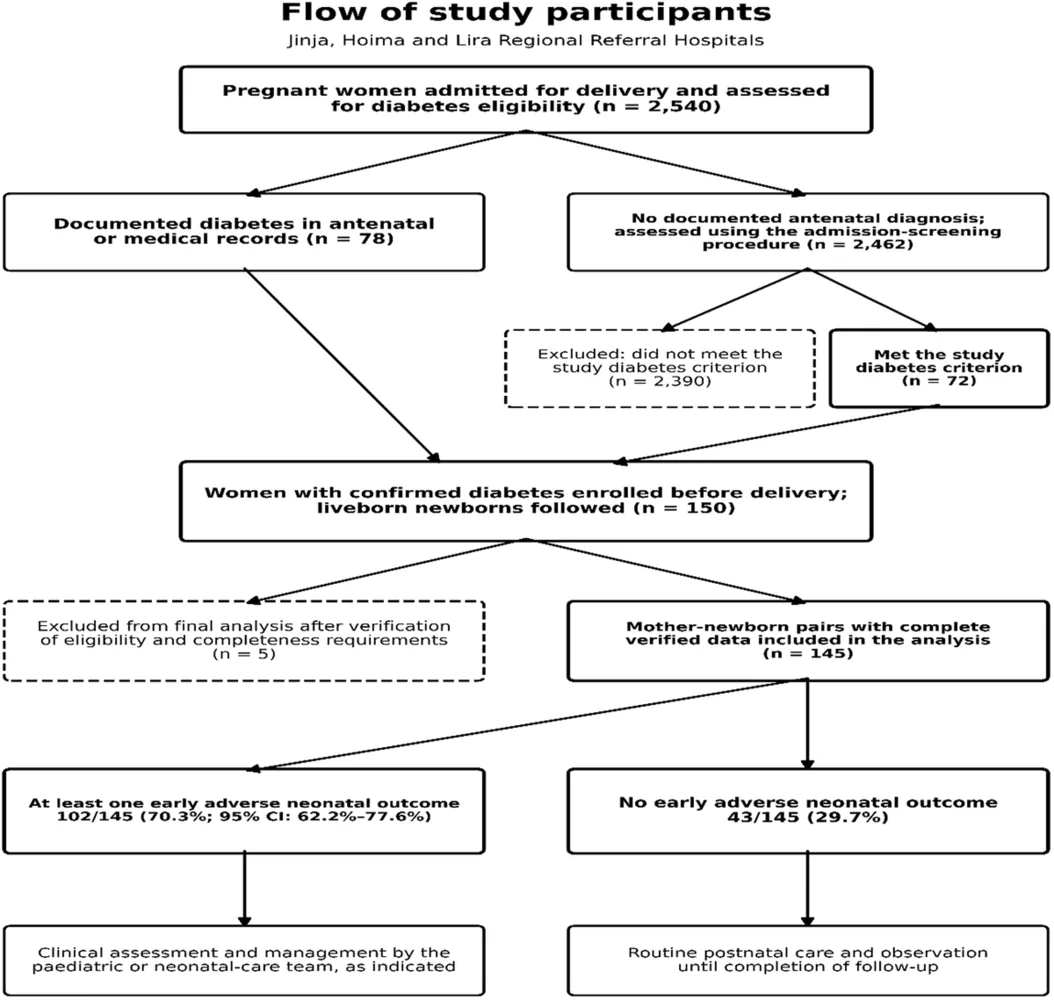
Study flow chart.

Among the 145 pregnant women with diabetes mellitus, 99/145 (68.3%) were aged 20–34 years, while 39/145 (26.9%) were aged ≥35 years. Sixty participants (41.4%) were overweight, and 27/145 (18.6%) were obese. Most participants were Christian (123/145; 84.8%), had attained secondary education (63/145; 43.4%), and resided in rural areas (80/145; 55.2%). At least four antenatal‐care contacts were reported by 113/145 participants (77.9%), and 101/145 (69.7%) reported exercising during pregnancy (Table 1).

**Table 1:** Baseline characteristics of pregnant women with diabetes mellitus (*N* = 145).

Variable	Category	Frequency, *n*	Percentage, %
Age, years	< 20	7	4.8
	20–34	99	68.3
	≥ 35	39	26.9
Body mass index, kg/m^2^	< 25.0	58	40.0
	25.0–29.9	60	41.4
	≥ 30.0	27	18.6
Religion	Christian	123	84.8
	Other	22	15.2
Education level	No formal education	10	6.9
	Primary	53	36.6
	Secondary	63	43.4
	Tertiary	19	13.1
Residence	Urban	65	44.8
	Rural	80	55.2
Antenatal‐care contacts	< 4	32	22.1
	≥ 4	113	77.9
Exercise during pregnancy	Yes	101	69.7
	No	44	30.3

*Note:* Percentages were calculated using 145 participants as the denominator. BMI was calculated as weight in kilograms divided by height in meters squared.

### 3.2 Incidence of Early Adverse Neonatal Outcomes

Among the 145 neonates born to women with diabetes mellitus, 102 developed at least one early adverse neonatal outcome, corresponding to a cumulative incidence of 70.3% (95% CI: 62.2%–77.6%). The remaining 43/145 neonates (29.7%) did not experience any component of the composite outcome (Figure 2). The three hospitals made comparable contributions to the study population. Lira Regional Referral Hospital enrolled 52/145 mother–newborn pairs (35.9%), Jinja Regional Referral Hospital enrolled 47/145 (32.4%), and Hoima Regional Referral Hospital enrolled 46/145 (31.7%). The composite early adverse neonatal outcome occurred in 38/52 neonates at Lira (73.1%; 95% CI 59.7%–83.2%), 34/46 at Hoima (73.9%; 95% CI 59.7%–84.4%), and 30/47 at Jinja (63.8%; 95% CI 49.5%–76.0%). There was no clear evidence that the proportion experiencing the composite outcome differed across the three hospitals (Pearson *χ* ^2 ^= 1.42, degrees of freedom = 2; *p* = 0.49).

**Figure 2:**
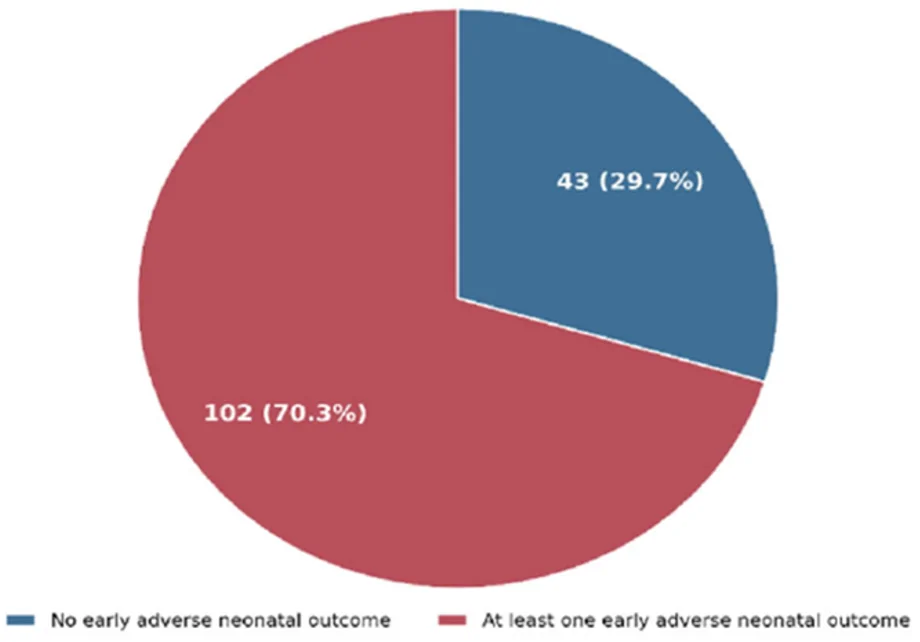
Cumulative incidence of early adverse neonatal outcomes among neonates born to women with diabetes mellitus (*N* = 145).

The composite outcome comprised macrosomia in 55/145 neonates (37.9%), a 5‐min Apgar score <7 in 51/145 (35.2%), neonatal hypoglycemia in 38/145 (26.2%), respiratory distress in 31/145 (21.4%) and early neonatal death in 21/145 (14.5%). These components were not mutually exclusive because a neonate could experience more than one outcome. NICU admission occurred in 40/145 neonates (27.6%) but was reported separately and was not included in the composite outcome (Figure 3).

**Figure 3:**
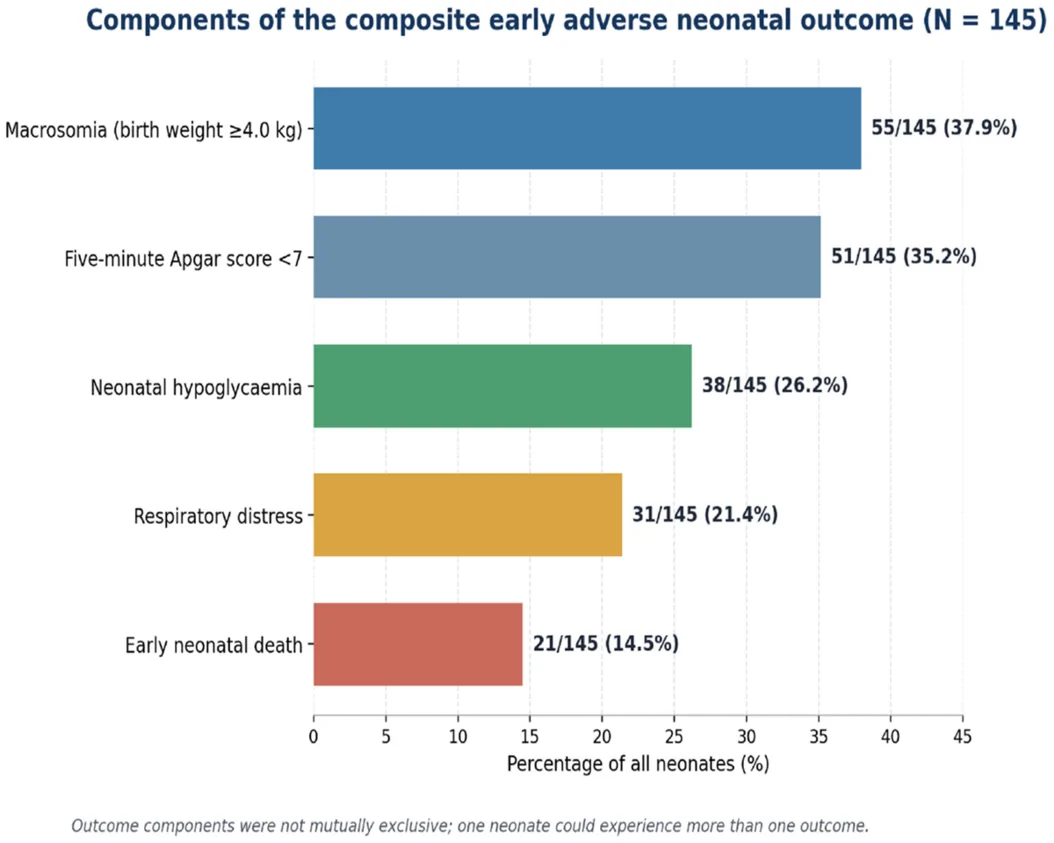
Distribution of individual early adverse neonatal outcomes among neonates born to women with diabetes mellitus (*N* = 145).

### 3.3 Factors Associated With Early Adverse Neonatal Outcomes Among Neonates Born to Women With Diabetes Mellitus

At bivariable analysis, maternal body mass index, residence, number of antenatal‐care contacts, and exercise during pregnancy had at least one category with *p*< 0.20 and were considered for multivariable analysis (Table 2). These four variables contributed five predictor coefficients to the final modified Poisson regression model, providing 20.4 adverse outcomes per parameter and limiting the risk of overfitting.

**Table 2:** Bivariable analysis of factors associated with the composite early adverse neonatal outcome (*N* = 145).

Variable	Category	No adverse outcome, *n* (%)	Adverse outcome, *n* (%)	cRR (95% CI)	*p* value
Age, years	20–34	27 (27.3)	72 (72.7)	1.00	—
	< 20	4 (57.1)	3 (42.9)	0.59 (0.25–1.40)	0.23
	≥ 35	12 (30.8)	27 (69.2)	0.95 (0.75–1.21)	0.69
Body mass index, kg/m^2^	< 25.0	15 (25.9)	43 (74.1)	1.00	—
	25.0–29.9	23 (38.3)	37 (61.7)	0.83 (0.65–1.07)	0.15
	≥ 30.0	5 (18.5)	22 (81.5)	1.10 (0.87–1.39)	0.43
Religion	Christian	38 (30.9)	85 (69.1)	1.00	—
	Other	5 (22.7)	17 (77.3)	1.12 (0.87–1.44)	0.39
Education level	Secondary	15 (23.8)	48 (76.2)	1.00	—
	No formal education	4 (40.0)	6 (60.0)	0.79 (0.47–1.33)	0.37
	Primary	17 (32.1)	36 (67.9)	0.89 (0.71–1.12)	0.33
	Tertiary	7 (36.8)	12 (63.2)	0.83 (0.57–1.20)	0.32
Residence	Urban	23 (35.4)	42 (64.6)	1.00	—
	Rural	20 (25.0)	60 (75.0)	1.16 (0.93–1.45)	0.18
Antenatal‐care contacts	≥ 4	30 (26.5)	83 (73.5)	1.00	—
	< 4	13 (40.6)	19 (59.4)	0.81 (0.59–1.10)	0.17
Exercise during pregnancy	Yes	22 (21.8)	79 (78.2)	1.00	—
	No	21 (47.7)	23 (52.3)	0.67 (0.49–0.90)	0.009

*Note:* Values are *n* (%); percentages are calculated within outcome groups. The composite outcome included macrosomia, 5‐min Apgar score < 7, neonatal hypoglycemia, respiratory distress, or early neonatal death. cRRs were estimated using bivariable modified Poisson regression with robust standard errors. Reference categories are indicated by 1.00.

Abbreviations: ANC, antenatal care; BMI, body mass index; CI, confidence interval; cRR, crude risk ratio.

After adjustment, maternal body mass index, residence, and number of antenatal‐care contacts were not independently associated with the composite early adverse neonatal outcome. Women who reported no exercise during pregnancy had a lower observed risk of the composite outcome than those who reported exercising (aRR 0.68, 95% CI 0.51–0.91; *p*= 0.009; Table 3).

**Table 3:** Multivariable analysis of maternal factors associated with the composite early adverse neonatal outcome among newborns of women with diabetes mellitus (*N* = 145).

Variable	Category	aRR	95% CI	*p* value
Maternal BMI (kg/m^2^)	< 25	1.00	Reference	—
	25.0–29.9	0.81	0.64–1.03	0.09
	≥ 30	1.07	0.85–1.36	0.55
Residence	Urban	1.00	Reference	—
	Rural	1.12	0.90–1.41	0.30
Antenatal‐care contacts	≥ 4	1.00	Reference	—
	< 4	0.87	0.65–1.17	0.35
Exercise during pregnancy	Yes	1.00	Reference	—
	No	0.68	0.51–0.91	0.009

*Note:* aRRs were estimated using multivariable modified Poisson regression with robust standard errors. The model included BMI, residence, ANC contacts, and self‐reported exercise. Reference categories are indicated by 1.00. All *p* values are two‐sided.

Abbreviations: ANC, antenatal care; aRR, adjusted risk ratio; BMI, body mass index; CI, confidence interval.

The inverse direction of this association was unexpected and should be interpreted cautiously. Exercise was self‐reported, and information on its type, frequency, intensity, and timing was unavailable. The estimate may therefore reflect exposure misclassification, differences in maternal health status, or residual confounding by diabetes severity, glycemic control, treatment, and pregnancy complications. It should not be interpreted as evidence that avoiding exercise during pregnancy reduces the risk of adverse neonatal outcomes.

## 4 Discussion

In this multicentre prospective cohort, 102/145 newborns of women with diabetes experienced at least one early adverse neonatal outcome, corresponding to a cumulative incidence of 70.3% (95% CI: 62.2%–77.6%). This substantial burden is biologically plausible because maternal hyperglycemia promotes fetal hyperinsulinemia, excessive fetal growth, and postnatal metabolic instability, increasing the risks of macrosomia, neonatal hypoglycemia, respiratory morbidity, and impaired neonatal adaptation [2, 3].

Our estimate is comparable to findings from the MOMPOD trial, in which composite adverse neonatal outcomes occurred in 71% of participants receiving metformin plus insulin and 74% receiving insulin plus placebo [20]. These similarly high estimates may reflect the inclusion of women with pre‐existing type 2 diabetes or diabetes diagnosed early in pregnancy, resulting in prolonged fetal exposure to hyperglycemia. Lower incidences have been reported in Northwest Ethiopia, where 51.7% of newborns of women with gestational diabetes mellitus experienced a composite adverse outcome, and among small‐for‐gestational‐age newborns exposed to gestational diabetes, among whom the corresponding incidence was 53.7% [19, 21]. Simmons et al reported incidences of 24.9% following immediate treatment of early gestational diabetes and 30.5% following deferred or no immediate treatment [15]. Differences between studies may reflect variations in diabetes type and severity, timing of diagnosis, glycemic control, treatment, referral patterns, duration of neonatal follow‐up, and the components included in composite outcomes. The comparatively high incidence in the present study may also reflect recruitment from regional referral hospitals that manage women with complicated diabetes, delayed referrals, and coexisting obstetric conditions.

Macrosomia was the most frequent component of the composite outcome, affecting 55/145 newborns (37.9%). Maternal glucose crosses the placenta and stimulates fetal insulin production, promoting fat deposition and accelerated fetal growth [2, 3]. This finding is consistent with evidence from Ethiopia and large international studies demonstrating increased risks of macrosomia and large‐for‐gestational‐age birth among women with gestational diabetes [7, 8, 19]. The occurrence of macrosomia may also be influenced by the duration and severity of maternal hyperglycemia, gestational weight gain, and glycemic control during late pregnancy. Because serial glucose measurements and glycated hemoglobin were unavailable, the contribution of these factors could not be evaluated.

A 5‐min Apgar score below 7 occurred in 51/145 newborns (35.2%). Although a low Apgar score may indicate impaired neonatal adaptation, it is not specific to maternal diabetes and may also reflect prematurity, intrapartum compromise, difficult delivery, congenital abnormalities, or the effects of obstetric interventions. Muche et al. similarly reported an increased risk of a low 5‐min Apgar score among newborns exposed to gestational diabetes [19]. The comparatively high proportion in the present cohort may reflect coexisting obstetric complications, differences in intrapartum care, and the high‐risk referral population. This finding should therefore be interpreted as a clinical indicator of neonatal condition rather than direct evidence of diabetes‐related hypoxia.

Neonatal hypoglycemia occurred in 38/145 newborns (26.2%). Following delivery, interruption of the maternal glucose supply in the presence of persistent fetal hyperinsulinemia may cause a rapid decline in neonatal glucose concentration [2, 3]. This finding reinforces the importance of early feeding, glucose monitoring, and prompt treatment among newborns of women with diabetes. Maternal glycemic control and the glucose‐lowering treatment received during pregnancy may also influence neonatal hypoglycemia risk [5, 16].

Respiratory distress occurred in 31/145 newborns (21.4%). Maternal diabetes has been associated with increased neonatal respiratory morbidity, including among infants delivered at or near term [9]. Fetal hyperinsulinemia may interfere with surfactant production and pulmonary maturation, providing a possible biological explanation for this association [3, 9]. A large French cohort also reported increased respiratory morbidity among pregnancies complicated by gestational diabetes [7]. Nevertheless, gestational age, mode of delivery, congenital abnormalities, and intrapartum complications may also have contributed to respiratory distress in the present cohort.

Early neonatal death occurred in 21/145 newborns (14.5%). This substantial proportion warrants cautious interpretation because neonatal death may result from interacting maternal, obstetric, and neonatal conditions and cannot be attributed to maternal diabetes alone. The inability to account adequately for gestational age, congenital anomalies, birth complications, and severity of neonatal illness further limits causal interpretation. Nevertheless, the observed burden emphasizes the need for appropriate delivery planning, skilled neonatal resuscitation, and early recognition and treatment of metabolic and respiratory complications.

Neonatal‐unit admission occurred in 40/145 newborns (27.6%) and was analyzed separately because admission is a healthcare‐utilization indicator rather than an adverse neonatal condition in itself. Admission may be influenced by neonatal illness, local clinical protocols, bed availability, and clinicians' thresholds for observing newborns exposed to maternal diabetes. It should therefore not be interpreted independently as evidence of biological neonatal morbidity.

Overall, fetal overgrowth, impaired neonatal adaptation, hypoglycemia, and respiratory morbidity contributed substantially to the composite outcome. Because individual outcomes were not mutually exclusive, their percentages should not be added. Comparisons across studies should also remain cautious because composite estimates depend on the number and definition of included components, diabetes type, diagnostic criteria, glycemic management, and duration and intensity of neonatal surveillance. Nevertheless, the observed distribution supports coordinated antenatal glycemic management, delivery planning, and immediate neonatal surveillance. Facilities managing women with diabetes should be prepared to monitor neonatal glucose concentrations, support respiratory transition, and provide timely neonatal resuscitation.

After adjustment, self‐reported exercise status was the only factor associated with the composite outcome. Women who reported no exercise had a 32% lower risk of an early adverse neonatal outcome than those who reported exercising (adjusted risk ratio [aRR] 0.68, 95% CI: 0.51–0.91; *p* = 0.009). The unexpected direction of this association should not be interpreted as evidence that avoiding exercise improves neonatal outcomes.

Available evidence generally indicates that appropriate physical activity during pregnancy improves maternal glucose regulation and may reduce the risk of gestational diabetes without increasing adverse neonatal outcomes [11, 12]. The inverse association observed in this study may therefore reflect residual confounding, exposure misclassification, reverse causation, or confounding by indication. For example, women with poorly controlled diabetes or recognized risk factors may have been advised to exercise after diagnosis, whereas women with obstetric complications may have restricted their activity. Exercise was measured as a binary self‐reported exposure without information on its type, frequency, intensity, duration, or timing. The finding should consequently be considered exploratory and requires confirmation using validated physical‐activity measurements.

Maternal body mass index was not clearly associated with the composite outcome after adjustment. Compared with women with a BMI below 25 kg/m^2^, the adjusted risk was lower but imprecisely estimated among women who were overweight (aRR 0.81, 95% CI: 0.64–1.03; *p* = 0.09) and was not clearly different among women with obesity (aRR 1.07, 95% CI: 0.85–1.36; *p* = 0.55). These findings differ from systematic‐review evidence associating elevated pre‐pregnancy BMI with macrosomia, low 5‐min Apgar scores, and other adverse neonatal outcomes [10, 22]. The discrepancy may reflect limited precision, differences between pre‐pregnancy and antenatal BMI, limited variation within a cohort composed entirely of women with diabetes, or residual confounding. The confidence intervals do not exclude potentially important associations.

Rural residence was also not clearly associated with the composite outcome (aRR 1.12, 95% CI: 0.90–1.41; *p* = 0.30). Although geographic residence may influence access to diabetes screening, referral and specialist care, residence alone may not adequately represent travel barriers, socioeconomic circumstances, or the quality and timeliness of care received.

Similarly, fewer than four antenatal‐care contacts were not clearly associated with the composite outcome when compared with four or more contacts (aRR 0.87, 95% CI: 0.65–1.17; *p* = 0.35). This estimate should not be interpreted as evidence that fewer contacts are beneficial or equivalent because its confidence interval includes both lower and higher risks. The number of contacts may also inadequately represent the timing, content, and quality of diabetes care, including glucose monitoring, nutritional counseling, treatment adherence, and referral to specialist services.

The limited number of clearly associated factors may reflect the modest sample size, high outcome incidence, and limited variability within a cohort composed entirely of women with diabetes. Important determinants, including diabetes type, timing of diagnosis, serial glucose concentrations, glycated hemoglobin, treatment adherence, gestational age at delivery, congenital abnormalities, and obstetric complications were unavailable or insufficiently measured. Because the severity and pattern of maternal hyperglycemia are important determinants of neonatal risk, residual confounding remains likely [5, 6]. The reported associations should therefore be considered exploratory and should not be interpreted as causal effects or as a validated clinical prediction model.

### 4.1 Strengths and Limitations

This prospective multicentre cohort provides evidence on early neonatal outcomes among women with diabetes in pregnancy receiving care at three regional referral hospitals in Uganda. Maternal characteristics were recorded before delivery, and newborns were followed prospectively, establishing the temporal sequence between the measured maternal factors and neonatal outcomes. Inclusion of hospitals from different regions increased the clinical and geographical diversity of the cohort. Complete early neonatal‐outcome data were available for all 145 mother–newborn pairs. The study reported both the cumulative incidence and distribution of clinically important outcomes, while neonatal‐unit admission was analyzed separately as a healthcare‐utilization indicator rather than as a neonatal complication. Because the composite outcome was common, modified Poisson regression with robust standard errors was used to estimate risk ratios and corresponding 95% confidence intervals.

Several limitations should be considered. First, the study was conducted in regional referral hospitals, which are likely to manage women with complicated diabetes, delayed referrals, and coexisting obstetric conditions. The observed incidence may therefore be higher than that in lower‐level facilities or the general population of women with diabetes in pregnancy, limiting generalizability. The absence of a comparison group of women without diabetes also means that the excess neonatal risk attributable specifically to maternal diabetes could not be estimated.

Second, although the study included three hospitals, clinical practices related to diabetes screening, glycemic management, timing of delivery, neonatal glucose monitoring, resuscitation, and neonatal‐unit admission may have differed between sites. Standardized study definitions and data‐collection procedures were used to improve comparability. Site‐specific outcome proportions were described; however, participant‐level hospital information was not available for inclusion in the adjusted regression model. Consequently, clustering and residual confounding by hospital‐level practices could not be fully addressed.

Third, diabetes classification relied primarily on diagnoses documented during routine antenatal or medical care. Previously unscreened women with classic symptoms and random blood glucose ≥200 mg/dL were classified as having overt diabetes, whereas those with values of 140–199 mg/dL alone were not enrolled. Because diagnostic methods were not standardized and oral glucose‐tolerance tests, glycated hemoglobin and longitudinal glycemic data were not consistently documented, misclassification of diabetes type or severity remains possible.

Fourth, the modest sample size limited statistical precision and the number of predictors that could be included safely in the multivariable model. Some clinically important associations may therefore have remained undetected. Important maternal, obstetric and neonatal characteristics, including congenital anomalies, detailed intrapartum complications and severity of neonatal illness could not be adequately controlled. Residual and unmeasured confounding remain likely, and the reported associations should not be interpreted as causal or as constituting a validated prediction model.

Fifth, exercise was self‐reported as a binary exposure without information on its type, frequency, intensity, duration, or timing. It was therefore susceptible to recall bias, social‐desirability bias, and exposure misclassification. Reverse causation and confounding by indication are also possible because women with poorly controlled diabetes may have been advised to exercise, whereas those with obstetric complications may have restricted their activity. The unexpected association between reporting no exercise and a lower risk of adverse outcomes should consequently be considered exploratory and does not provide evidence that women with diabetes should avoid appropriate physical activity.

Sixth, the composite outcome combined events that differ in severity, biological mechanism, and clinical importance. Its components were not mutually exclusive, and its high incidence was partly influenced by the number and frequency of the included outcomes. A 5‐min Apgar score below 7 is a general measure of neonatal condition and is not specific to diabetes‐related fetal compromise. Similarly, macrosomia may be influenced by maternal adiposity, gestational weight gain, and other factors in addition to hyperglycemia. Neonatal‐unit admission was excluded from the composite and reported separately because it may be influenced by local protocols, bed availability, and clinicians' admission thresholds.

Finally, follow‐up was limited to the early neonatal period. The study could not assess subsequent readmission, later neonatal mortality, growth, or long‐term neurodevelopmental outcomes. Larger prospective studies using standardized diagnostic criteria, objective measures of glycemic control, harmonized clinical protocols, adjustment for hospital‐level clustering, and longer neonatal follow‐up are needed to confirm these findings.

## 5 Conclusions and Recommendations

Early adverse neonatal outcomes were common among newborns of women with diabetes in pregnancy, affecting approximately 7 in 10 newborns. Macrosomia, a 5‐min Apgar score below 7, neonatal hypoglycemia, and respiratory distress contributed substantially to this burden. Neonatal‐unit admission was also frequent, but represents healthcare utilization rather than a neonatal complication in itself.

Reported exercise status was the only factor independently associated with the composite outcome. However, the unexpected lower risk among women reporting no exercise may reflect exposure misclassification, reverse causation, confounding by indication, or residual confounding. It should not be interpreted as evidence that avoiding exercise improves neonatal outcomes. Maternal body mass index, residence, and antenatal‐care attendance were not clearly associated with the composite outcome, although the confidence intervals did not exclude potentially important effects.

Health facilities managing women with diabetes should strengthen antenatal glycemic monitoring, individualized delivery planning, and preparedness for early neonatal glucose assessment, respiratory support, and resuscitation. Appropriate physical activity should continue to be promoted according to clinical guidance and individual obstetric circumstances.

Larger multicentre prospective studies are recommended to confirm these findings using standardized diagnostic criteria for diabetes, objective measurements of glycemic control and physical activity, harmonized outcome definitions, and adjustment for hospital‐level differences. Future studies should also evaluate individual neonatal outcomes separately and extend follow‐up beyond the early neonatal period. The present associations should be considered exploratory and should not be interpreted as causal or as a validated clinical prediction model.

## Author Contributions

**Sharifah Namutebi:** conceptualization, methodology, software. **Marie Pascaline Sabine Ishimwe:** conceptualization, software, writing – original draft. **Musa Kasujja:** investigation, writing – original draft, formal analysis. **Theodore Nteziyaremye:** investigation, methodology, software, visualization. **Silver Bagambe:** conceptualization, methodology, investigation. **Ibrahim Bwaga:** methodology, writing – original draft, investigation. **Adam Damulira:** investigation, methodology, supervision. **Peter Okello:** investigation, funding acquisition, validation, data curation. **Asanairi Baluku:** investigation, writing – review and editing, visualization. **Theoneste Hakizimana:** conceptualization, methodology, software, formal analysis, writing – review and editing.

## Funding

The authors have nothing to report.

## Conflicts of Interest

The authors declare no conflicts of interest.

## Transparency Statement

Theoneste Hakizimana, the manuscript guarantor, affirms that this manuscript is an honest, accurate, and transparent account of the study being reported; that no important aspects of the study have been omitted; and that any discrepancies from the study as planned have been explained.

## Data Availability

The deidentified data supporting the findings of this study are available from the corresponding author upon reasonable request, subject to applicable ethical and institutional requirements. Please contact Theoneste Hakizimana via email at theonestehakizimana5@gmail.com.
